# Targeting Oxidative Stress and Inflammation in Intervertebral Disc Degeneration: Therapeutic Perspectives of Phytochemicals

**DOI:** 10.3389/fphar.2022.956355

**Published:** 2022-07-12

**Authors:** Liang Kang, Huaqing Zhang, Chongyu Jia, Renjie Zhang, Cailiang Shen

**Affiliations:** Department of Orthopedics and Spine Surgery, The First Affiliated Hospital of Anhui Medical University, Hefei, China

**Keywords:** intervertebral disc degeneration, oxidative stress, inflammation, phytochemicals, therapeutic implication

## Abstract

Low back pain is a major cause of disability worldwide that declines the quality of life; it poses a substantial economic burden for the patient and society. Intervertebral disc (IVD) degeneration (IDD) is the main cause of low back pain, and it is also the pathological basis of several spinal degenerative diseases, such as intervertebral disc herniation and spinal stenosis. The current clinical drug treatment of IDD focuses on the symptoms and not their pathogenesis, which results in frequent recurrence and gradual aggravation. Moreover, the side effects associated with the long-term use of these drugs further limit their use. The pathological mechanism of IDD is complex, and oxidative stress and inflammation play an important role in promoting IDD. They induce the destruction of the extracellular matrix in IVD and reduce the number of living cells and functional cells, thereby destroying the function of IVD and promoting the occurrence and development of IDD. Phytochemicals from fruits, vegetables, grains, and other herbs play a protective role in the treatment of IDD as they have anti-inflammatory and antioxidant properties. This article reviews the protective effects of phytochemicals on IDD and their regulatory effects on different molecular pathways related to the pathogenesis of IDD. Moreover, the therapeutic limitations and future prospects of IDD treatment have also been reviewed. Phytochemicals are promising candidates for further development and research on IDD treatment.

## Introduction

Low back pain (LBP) is one of the most prevalent musculoskeletal disorders in the world, and it is estimated that nearly 80% of the population suffers from LBP during their lifetime. The occurrence of LBP in a patient induces a severe burden on their families and society. Intervertebral disc (IVD) degeneration (IDD) is currently believed to be an essential cause of LBP, and it forms the pathophysiological basis of several spinal degenerative diseases, such as intervertebral disc herniation and spinal stenosis (Wang et al., 2014; Vo et al., 2016; Oichi et al., 2020). The conservative clinical treatment for the early stages of IDD primarily includes nonsteroidal anti-inflammatory drugs, physical therapy, and rest. However, such treatment is limited to reducing or controlling pain and does not reverse the process of IDD. Moreover, the long-term use of nonsteroidal anti-inflammatory drugs has apparent side effects (Conaghan, 2012; Bindu et al., 2020). If IDD develops to an advanced stage, surgical treatment, including discectomy and interbody fusion, is required. Although surgical treatment is recommended to be effective for IDD, it is expensive and is associated with several complications, such as adjacent segment disease (Lau et al., 2021), decreased spinal mobility, and limited function. Hence, it is imperative to explore new methods for the treatment of IDD.

The IVD, which is the largest avascular structure, is an important component of the load-bearing capacity of the spine. It comprises three distinct regions: the centrally located nucleus pulposus (NP), peripheral annulus fibrosus (AF) surrounding NP, and cartilage endplate (CEP) located above and below (Adams and Roughley, 2006). The NP is composed mainly of water and a rich extracellular matrix (ECM), which provides resistance to IVD against axial pressures that are transmitted down the spine. The AF consists of concentrically arranged fibrous layers (15–25 layers) that resist the lateral expansion of the IVD during weight-bearing activities. The CEP not only seals the IVD, but also attaches it to the vertebral body. Most importantly, CEP furnishes a permeable barrier between the IVD and the vertebral body to provide nutrition for the IVD cells, but its ability to provide nutrition is limited. Therefore, it is difficult for IVD to repair itself in case of injury (Freemont, 2009).

The presence of three distinct anatomical regions generates the structural complexity of the disc. Healthy discs transmit and absorb stresses in the spine and maintain the multi-axial flexibility of the spine. In contrast, degenerating discs show structural damage that is characterized by high disc collapse, AF rupture, NP tissue loss, decreased water content, and CEP calcification (Ding et al., 2013). IDD is a multifactorial disease with etiologies including infection, genetic susceptibility, aging, trauma, smoking, and diabetes. Many signaling pathways and effector molecules have been implicated in the IDD process, and elucidation of the pathological mechanisms of IDD will facilitate the improvement of its treatment options. Recently, a growing number of studies have revealed a close relationship between inflammation, oxidative stress, and the incidence of IDD (Dowdell et al., 2017). Therefore, antioxidant and anti-inflammatory treatments have been proposed as promising strategies for the treatment of IDD. The following section details the roles of inflammation and oxidative stress in the pathogenesis of IDD.

## Inflammation in Intervertebral Disc Degeneration

Increasing evidence has shown that inflammation is implicated in the occurrence and development of IDD. Several pro-inflammatory cytokines, such as IL-6, IL-17, IL-1α, TNF-α, IL-1β, and IL-8, significantly contribute to an increase in degenerative IVD. These cytokines are closely associated with several key pathophysiological processes of IDD (Johnson et al., 2015; Wang Y. et al., 2020). IL-1β stimulation has been reported to significantly enhance the expression of IL-6, IL-8, and IL-17 in human IVD cells, resulting in an inflammatory cascade. Simultaneously, a feedback loop is formed between these pro-inflammatory cytokines, which forms a persistent local inflammatory microenvironment (Jimbo et al., 2005; Jia et al., 2020). Furthermore, the balance between catabolism and anabolism of ECM is essential for maintaining the structural and functional integrity of IVD, i.e., if the ECM catabolic activity is higher than anabolic activity, it can result in the occurrence of IDD. The primary enzymes that cleave ECM components include matrix metalloproteinases (MMPs) and a disintegrin and metalloprotease with thrombospondin motifs (ADAMTS) (Le Maitre et al., 2007; Vo et al., 2013). Some ECM-degrading enzymes, such as MMP-1/3/7/9 and ADAMTS-1/4/5/9 are significantly increased in degenerative IVD (Le Maitre et al., 2007). Furthermore, pro-inflammatory cytokines can contribute to ECM degradation and the subsequent destruction of the IVD structure by promoting the expression of ECM-degrading enzymes. Apoptosis refers to the spontaneous and orderly death of cells regulated by genes in order to maintain tissue homeostasis. Under physiological conditions, apoptosis plays an important role in maintaining tissue homeostasis. However, in the pathological state, excessive apoptosis stimulated by risk factors will lead to a significant reduction in the number of IVD cells, resulting in the destruction of the structure and function of IVD. Notably, several studies have shown that pro-inflammatory cytokines can induce apoptosis of IVD cells, and then lead to the occurrence and development of IDD. Cell senescence is an irreversible cell cycle arrest caused by a number of factors, such as oxidative stress, pro-inflammatory cytokines, DNA damage. Senescent cells are active and exhibit pro-inflammatory and catabolic phenotypes. Pro-inflammatory cytokines can also accelerate the senescence of IVD cells. Senescent cells can produce more matrix-degrading enzymes and pro-inflammatory cytokines, resulting in further deterioration of the IVD microenvironment (Zhang et al., 2020). Recently, the relationship between inflammation and oxidative stress has also attracted much attention. Pro-inflammatory cytokines have been proved to promote the excessive production of ROS in IVD cells, and then induce oxidative injury of IVD cells (Wang Y. et al., 2020). Vascular endothelial growth factor (VEGF) is an essential member of the pro-angiogenic factor. The expression of VEGF in degenerative IVD is significantly upregulated. Studies have revealed that pro-inflammatory cytokines can upregulate the expression of VEGF in IVD (Kwon et al., 2017). Most importantly, these pro-inflammatory factors can induce stimulation of sinus vertebral nerve endings (Ohtori et al., 2012a; Ohtori et al., 2012b). These nerve endings grow into the IVD and cause nerve root pain (Freemont et al., 2002; Orita et al., 2010), which is the main cause of chronic LBP (Johnson et al., 2001). These findings highlight that inflammation has a central role in the pathogenesis of IDD (Francisco et al., 2022) and suggest that anti-inflammatory strategies can be promising for the treatment of IDD.

## Oxidative Stress in Intervertebral Disc Degeneration

**FIGURE 1:**
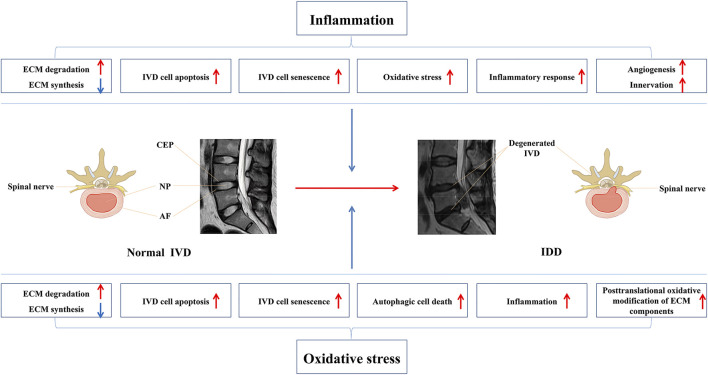
Inflammation and oxidative stress are involved in multiple pathological processes of IDD.

Reactive oxygen species (ROS) are unstable and highly reactive molecules (Feng et al., 2017). They include superoxide anions (O_2_^−^), hydrogen peroxide (H_2_O_2_), hydroxyl radicals (OH^−^), and hypochlorite ions (OCL^−^) (Cao et al., 2022). They are the by-products of oxidative metabolism (Reuter et al., 2010; Kim and Yim, 2015). Although the nutrition supply of IVD is low, the NP, AF, and CEP cells are not anaerobic (Bartels et al., 1998; Lee et al., 2007). During IDD, neovascularization increases the blood supply in IVD cells, thereby increasing the nutrient supply, which promotes ROS production from the original nutrient-deficient disc cells and results in oxidative stress (Gille and Nohl, 2001; Turrens, 2003). Oxidative stress is attributed to an imbalance between ROS production and the protective mechanism of antioxidants, resulting in molecular oxidative damage and cell destruction, which has adverse implications on the body. Studies have shown that ROS are widely involved in signal transduction, metabolic regulation, apoptosis, cell senescence, and the phenotypic transformation of cells in IDD (Feng et al., 2017; Cao et al., 2022). Excessive ROS activates the NF-κB and MAPK pathways, resulting in an imbalance between degradation and synthesis of ECM in disc cells and an increase in the secretion of pro-inflammatory factors. These changes eventually lead to the loss of disc cells and the persistence of the inflammatory microenvironment, which further leads to the destruction of IVD and the production of ROS (Zhou et al., 2010; Zhu et al., 2019). Autophagy is a protective process in which cells degrade metabolic waste and further reuse it; however continuous oxidative stress can induce excessive autophagy and lead to cell death (Chen et al., 2015; Filomeni et al., 2015; Cao et al., 2022). In addition, oxidative stress can destroy mitochondria and release pro-apoptotic molecules from the mitochondria into the cytoplasm, causing cascade reactions and cell apoptosis (Chen et al., 2014; Yang et al., 2015). Excessive ROS production can also promote IVD cell senescence, thus promoting the secretion of pro-inflammatory factors, leading to adjacent IVD cell senescence, apoptosis, and ECM degradation (Dimozi et al., 2015). Moreover, the increase of ROS production leads to the triggering of glycosylation reaction which further results in the increase of endogenous active by-products and the generation of advanced glycation end products (AGEs) (Vistoli et al., 2013). AGEs have been proved to be closely related to the pathogenesis of IDD. They can promote the inflammation, apoptosis, and ECM degradation of IVD cells, resulting in the destruction of IVD structure and function (Song et al., 2017; Song et al., 2018). These findings indicate that antioxidation is a new and effective treatment for IDD. Figure 1 provides an overview of the mechanisms by which inflammation and oxidative stress participate in the occurrence and development of IDD.

## Phytochemicals for the Treatment of IDD by Targeting Inflammation and Oxidative Stress

In recent years, several phytochemicals from traditional medicinal plants have been studied owing to their low cost, wide availability, and diverse biological activities. Owing to their anti-inflammatory and antioxidant properties, phytochemicals have been used to treat several diseases, such as myocardial ischemia, traumatic brain injury, osteoarthritis, and cancer (Cai et al., 2021; Deng et al., 2021; Tian et al., 2021; Chen C. et al., 2022). Several *in vivo* and *in vitro* experiments (Zhu et al., 2020; Chen et al., 2021) have shown that phytochemicals can play a protective role against IDD by targeting inflammation and oxidative stress. The effects and mechanisms of various phytochemicals against IDD are described in the following sections (Table 1).

**TABLE 1:** Phytochemicals possess multiple pharmacological effects *via* the anti-inflammatory and antioxidant mechanism in various *in vitro* and *in vivo* models of IDD.

Phytochemical	Study model	Dosage range	Signal pathways/Mechanisms	References
Curcumin	*In vitro*, TBHP induced human NP cells	5, 10, 15, 20, 25 μM	inhibited oxidative stress and mitochondrial dysfunction through AMPK/mTOR/ULK1 pathway-induced autophagy and -enhanced autophagic flux	Kang et al. (2019)
	*In vivo*, a needle puncture induced rat IDD model	100 mg/kg *via* intraperitoneal injection, twice weekly for 1 month		
Resveratrol	*In vitro*, SNP induced rat NP cells	10, 50, 100, 200, 400 μM	inhibited SNP-induced NP cell apoptosis by reducing ROS production	Li et al. (2018)
	*Ex vivo*, SNP induced organ culture of IVD	100 μM		
	*In vitro*, IL-1β induced rat NP cells	50 μM	reduced the expression levels of inflammatory factors in NP cells	Wuertz et al. (2011)
Mangiferin	*In vitro*, TNF-α induced human NP cells	100 μM, 500 μM	inhibited NP cell apoptosis and ECM degeneration by inhibiting the production of inflammatory factors and ROS; NF-κB signaling pathway	Yu et al. (2021)
	*In vivo*, a needle puncture induced rat IDD model	0.2 μg *via* intradiscal injection 3 days after puncture		
(-)-Epigallocatechin-3-gallate	*In vitro*, H_2_O_2_ induced human NP cells	5 μM, 25 μM	inhibited the expression level of inflammatory mediators and apoptosis; cGAS/Sting/NLRP3 pathway	Tian et al. (2020)
Chlorogenic acid	*In vitro*, IL-1β induced mice CEP cells	6.25, 12.5, 25 μM	inhibited the expression of inflammatory mediator and ECM degradation; NF-κB pathway	Ge et al. (2021)
Icariin	*In vitro*, IL-1β induced human NP cells	0.1, 1, 10 μM	inhibited the level of PGE2, NO, iNOS, COX-2 and ECM degradation; MAPK pathway and NF-κB pathway	Hua et al. (2018)
	*In vitro*, H_2_O_2_ induced human NP cells	0.1, 1, 10 μM	inhibited the production of ROS induced by H_2_O_2_; alle*via*ted the human NP cell apoptosis; Nrf2 pathway	Hua et al. (2020)
Lycopene	*In vitro*, H_2_O_2_ induced human NP cells	2.5 μm, 5 μm	Inhibited the apoptosis and ECM degradation; Nrf2 pathway	Lu et al. (2020)
Celastrol	*In vitro*, IL-1β induced human NP cells	10, 50, 100, 200 nM	Inhibited the apoptosis, ECM degradation, inflammation; NF-κB pathway	Chen et al. (2017)
Isofraxidin	*In vitro*, IL-1β induced human NP cells	10, 20, 40 μM	inhibited the level of PGE2, NO, iNOS, COX-2, TNF-α, and IL-6; NF-κB pathway	Su et al. (2019)
Higenamine	*In vitro*, IL-1β induced human NP cells	10, 20, 40 μM	inhibited the level of PGE2, iNOS, COX-2, TNF-α, and IL-6; NF-κB pathway	Bai et al. (2019)
Sesamin	*In vitro*, LPS induced rat NP cells	0.1, 0.5, 1 μM	suppressed the expression of inflammation factors and the migration of macrophages induced by LPS; MAPK pathway	Li et al. (2016)
	*Ex vivo*, LPS induced organ culture of IVD	10 μg/ml		
	*In vivo*, a needle puncture induced rat IDD model	0.1 M *via* intradiscal injection immediately after lesion of the disc		Li and Lv, (2020)
Honokiol	*In vitro*, H_2_O_2_ induced rat NP cells	2.5μM, 5 μM	inhibited the production of oxidative stress marker molecules (ROS, MDA) and the level of inflammatory mediators (IL-6, COX-2 and iNOS) in NP cells; NF-κB pathway, JNK signal, TXNIP/NLRP3/caspase-1/IL-1β signal axis	Tang et al. (2018)
	*In vivo*, a needle puncture induced rat IDD model	30 mg/kg *via* intraperitoneal injection, twice weekly for 2 month		
	*In vitro*, TBHP induced rat NP cells	1, 5, 10 μM	improved mitochondrial antioxidant capacity, mitochondrial function, and prevented oxidative stress in NP cells; AMPK-PGC-1α signaling pathway	Wang et al. (2018b)
	*In vivo*, a needle puncture induced rat IDD model	40 mg/kg *via* oral administration for 1 week		
Salvianolic acid B	*In vitro*, H_2_O_2_ induced rat NP cells	0.001, 0.01, 0.1, 1, 10, 100 nM	reduced the levels of ROS and MDA and increased the levels of GSH and SOD2	Dai et al. (2021b)
	*In vivo*, a needle puncture induced rat IDD model	20 mg/kg *via* oral gavage, once per day for six consecutive weeks		
Polydatin	*In vitro*, TNF-α induced rat NP cells	200 μM, 400 μM	reduced the production of ROS through Nrf2 signaling pathway	Wang et al. (2018a)
	*In vivo*, a needle puncture induced rat IDD model	50 mg/kg *via* intragastric administration, once per day for 4 weeks		
	*In vitro*, H_2_O_2_ induced human CEP cells	200 μM	upregulated Parkin and Nrf2 pathway, protecting CEP cells from H_2_O_2_-induced mitochondrial dysfunction, oxidative stress and apoptosis	Kang et al. (2020)
Naringin	*In vitro*, TNF-α induced human NP cells	5 μg/ml, 10 μg/ml, 20 μg/ml	prevented NP cells from inflammatory response, oxidative stress and impaired cellular homeostasis; AMPK/SIRT1 pathway	Chen et al. (2022b)
Baicalein	*In vitro*, IL-1β induced rat NP cells	5, 25, 50 μM	inhibited the level of NO, PGE2, TNF-α and IL-6 induced by IL-1β in NP cells; NF-κB and MAPK pathways	Jin et al. (2019)
	*In vivo*, a needle puncture induced rat IDD model	20 mg/kg *via* intraperitoneal injection, once daily		
Berberine	*In vitro*, H_2_O_2_ induced human NP cells	1, 2, 4, 8 μM	inhibited oxidative stress-induced cell damage by regulating ER stress and autophagy; IRE1/JNK pathway	Luo et al. (2019)
	*In vivo*, a needle puncture induced rat IDD model	150 mg/kg *via* intraperitoneal injection, once per day for 8 weeks		
	*In vitro*, IL-1β induced human NP cells	25 μM	inhibited inflammation-induced cell injury	Lu et al. (2019)
Genistein	*In vitro*, TBHP induced rat NP cells	50 μM, 100 μM	inhibited TBHP-induced apoptosis and ECM degradation; Nrf2 pathway	Wang et al. (2019)
	*In vivo*, a needle puncture induced rat IDD model	100 mg/kg/day via intragastric administration for 1 week before surgery		
Acacetin	*In vitro*, TBHP induced rat NP cells	0.3 μM, 1 μM	inhibited TBHP-induced ROS production in NP cells; reduced the expression of inflammatory mediators such as COX-2 and iNOS; Nrf2 and MAPK pathway	Wang et al. (2020b)
	*In vivo*, a needle puncture induced rat IDD model	25 mg/kg *via* intraperitoneal injection, once weekly for 4 months		
Wogonin	*In vitro*, IL-1β induced rat NP cells	10, 25, 50 μM	Inhibited IL-1β-induced inflammatory response and extracellular matrix degradation in NP cells	Fang et al. (2018)
Luteoloside	*In vitro*, IL-1β induced rat NP cells	2, 5, 10 μM	inhibited IL-1β-induced the level of NO, PGE2, TNF-α, IL-6, COX-2, and iNOS in rat NP cells; Nrf2/NF-κB pathway	Lin et al. (2019)
	*In vivo*, a needle puncture induced rat IDD model	10 mg/kg *via* intraperitoneal injection, once daily		
Quercetin	*In vitro*, TBHP induced rat NP cells	5, 15, 30, 60 μM	reduced ROS by activating SIRT1-autophagy pathway	Wang et al. (2020a)
	*In vivo*, a needle puncture induced rat IDD model	100 mg/kg *via* intraperitoneal injection, three times weekly for 8 weeks		

### Curcumin

Curcumin (CUR)—an active polyphenol extracted from the dried rhizomes of *Curcuma longa*—has been traditionally used for dietary and medical purposes worldwide (Li KX. et al., 2022; Jin et al., 2022; Ojo et al., 2022). CUR shows a wide range of pharmacological activities, including anti-inflammatory and antioxidant activities, in various disease models (Yang et al., 2020; Uddin et al., 2021; Fan and Lei, 2022). Kang et al. confirmed the protective effect of CUR against IDD using *in vivo* and *in vitro* experiments (Kang et al., 2019). CUR induces autophagy and enhanced autophagic flux *via* the AMPK pathway (Kang et al., 2019), thereby inhibiting oxidative stress and mitochondrial dysfunction. Moreover, the apoptosis, ECM degradation, and senescence were reversed by CUR in NP cells that were treated with tert-butyl hydroperoxide (TBHP). This study provides sufficient evidence suggesting that CUR delays IDD development by inhibiting oxidative stress.

### Resveratrol

Resveratrol (RES) is a polyphenolic compound present in many plants, such as berries and peanuts (Li KX. et al., 2021; Izzo et al., 2021). It has strong protective effects against many diseases, such as osteoarthritis and cancer (Mobasheri et al., 2012; Nguyen et al., 2017; Xiao et al., 2018; Deng et al., 2019; Cháirez-Ramírez et al., 2021). Li et al. found that RES protects rat NP cells from apoptosis induced by sodium nitroprusside by scavenging ROS in vitro experiments (Li et al., 2018). Moreover, *ex vivo* experiments showed that RES could reduce the development of experimental IDD. The secretion of pro-inflammatory cytokines by IVD cells appears to be the key mediator in the development of pain. Wuertz et al. found that RES significantly reduces the expression of IL-6 and IL-8 in NP cells (Wuertz et al., 2011). Therefore, RES may inhibit the progression of IDD through its dual effects of anti-inflammatory and antioxidant activities. Hence, it can be explored as a new method for IDD treatment.

### Mangiferin

Mangiferinis mainly extracted from *Mangifera persiciformis, Anemarrhena asphodeloides*, and *Mangifera indica* (Akter et al., 2022; Wang et al., 2022). It plays an important role in the progression of kidney disorders (Lum et al., 2022), diabetes (Aswal et al., 2020), cancer (Morozkina et al., 2021), and osteoarthritis (Qu et al., 2017; Li et al., 2019). It has crucial anti-inflammatory (Wang R. et al., 2021; Li N. et al., 2021) and antioxidant functions (Huang et al., 2020; Samadarsi and Dutta, 2020; Ismail et al., 2021); its application has also been reported in the treatment of IDD. Yu et al. found that mangiferin treatment can inhibit the loss of ECM by inhibiting TNF-α-induced inflammatory cytokines such as iNOS and COX-2 (Yu et al., 2021). In addition, it can alleviate mitochondrial damage and apoptosis indicators, such as cleaved-caspase-3 and Bax, by reducing ROS production. Moreover, *in vivo* experiments have confirmed the protective effect of mangiferin against IDD (Yu et al., 2021). These results suggest that mangiferin can provide a potential treatment for IDD.

### (-)-Epigallocatechin-3-Gallate

(-)-Epigallocatechin-3-gallate (EGCG) is a polyphenol that is abundantly found in tea (Butt et al., 2015). EGCG shows a variety of functions in many diseases, such as antiarteriosclerosis (Liu S. et al., 2017), antioxidant (Park et al., 2021), antibacterial (Noor Mohammadi et al., 2019), anti-inflammatory (Ma et al., 2021), and anti-tumor activities (Farabegoli and Pinheiro, 2022). The flow cytometry results of the study (Tian et al., 2020) conducted by Tian et al. showed that EGCG could inhibit the apoptosis and cell cycle arrest induced by H_2_O_2_. Western blot results showed that EGCG upregulates the anti-apoptotic proteins expression and downregulates the pro-apoptotic protein expression in H_2_O_2_-treated cells. Tian et al. analyzed the expression of IL-6,IL-1β, IL-10, and TNF-α in NP cells to explore the effect of EGCG on the NP cell’s inflammatory response. It was found that H_2_O_2_ could promote their expression, whereas EGCG could reverse the changes induced by H_2_O_2_. These results suggest that EGCG may be an alternative treatment for IDD.

### Chlorogenic Acid

Chlorogenic acid (CGA) is a natural biologically active compound that is abundantly present in coffee, fruits, and vegetables (Nwafor et al., 2022). It is also the main active ingredient in Chinese herbal medicines, such as *Honeysuckle* and *Eucommia*. Its biological functions in disease treatment, such as antioxidant, anti-inflammatory, and immune protective functions, have attracted considerable attention (Liang and Kitts, 2015; Bagdas et al., 2020; Kzhyshkowska, 2022). Ge et al. showed that CGA could reverse the downregulation of aggrecan, the main protein involved in the extracellular matrix anabolism of CEP cells induced by IL-1β, and inhibit the upregulation of MMP-13, the main protein involved in extracellular matrix catabolism (Ge et al., 2021). It can also inhibit the expression of inflammatory factors. Exploration of the molecular mechanisms revealed that the NF-κB signaling is the anti-degenerative effector molecule of CGA. Considering the important role of the NF-κB in the pathogenesis of IDD, these results suggest that CGA can reverse IDD development by regulating this pathway.

### Icariin

Icariin (ICA) is a flavonoid compound extracted from the widely known Chinese herbal medicine *Epimedium* (Wang M. et al., 2020), which is also recognized as “Yin Yang Huo”. ICA has been revealed by many studies to play a crucial role in antioxidation (Liu XJ. et al., 2021; Zheng et al., 2021) and anti-inflammatory (Guangtao et al., 2021; Zhang et al., 2022). Hua et al. found that IL-1β induces significant expression of COX-2 and iNOS and stimulates the production of PGE2 and nitric oxide in human NP cells (Hua et al., 2018). ICA usage can significantly reduce the levels of these inflammatory mediators. In addition, ICA reduces the expression levels of MMP-3/9/13 and ADAMTS-4/5 induced byIL-1β and increases the expression levels of type II collagen and aggrecan (Hua et al., 2018). A molecular mechanism study showed that MAPK and NF-κB are closely related to ICA. The research group also conducted relevant studies on oxidative stress. They found that ROS production increased in H_2_O_2_-treated human NP cells; however, this increase was inhibited by ICA in a dose-dependent manner (Hua et al., 2020). ICA can inhibit the mitochondrial cytochrome c translocation to cytoplasm, decrease Bax and caspase-3 levels, and increase Bcl-2 in H_2_O_2_-treated NP cells (Hua et al., 2020). The Nrf2 signaling pathway is an important member of the anti-oxidative stress system in cells. It has been shown to be involved in the antioxidant effects of ICA. Therefore, the study of ICA against inflammation and oxidative stress to maintain IVD cell homeostasis could prove the significance of ICA in the treatment of IDD.

### Lycopene

Lycopene is a naturally occurring, effective antioxidant found in reddish pink-colored fruits and vegetables, such as tomatoes (Mozos et al., 2018). The human body cannot synthesize lycopene and must be ingested through the diet. The powerful antioxidant effects of lycopene have attracted considerable attention and have been verified in many disease models (Müller et al., 2016; Chen et al., 2019; Imran et al., 2020). The upregulation of Bax and downregulation of Bcl-2 in H_2_O_2_-treated human NP cells were attenuated by lycopene (Lu et al., 2020). Flow cytometry also showed that lycopene inhibits NP cell apoptosis. In addition, lycopene can promote the expression of type II collagen, aggrecan, and Sox9, in NP cells. The molecular mechanism of lycopene involves Nrf2, which is a powerful antioxidant transcription factor closely related to the role of lycopene in H_2_O_2_-treated human NP cells. Therefore, lycopene has the potential to mediate antioxidation and treat IDD.

### Celastrol

Celastrolis a natural triterpenoid found in *Tripterygium wilfordii* that has been used to treat a variety of common diseases because of its strong anti-inflammatory activity (Jing et al., 2021; Zhu et al., 2021; Li M. et al., 2022). Celastrol can inhibit the upregulation of IL-6andTNF-α expression in NP cells induced by IL-1β (Chen et al., 2017). IL-1β can promote the MMP-3/9/13 and ADAMTS-4/5 expression. Celastrol inhibits the upregulation of these ECM-degrading enzymes. Moreover, since NF-κB acts as a crucial factor in promoting the inflammatory responses during IDD, celastrol can inhibit the activation of the NF-κB pathway (Chen et al., 2017). Therefore, celastrol has the potential for the treatment of IDD.

### Isofraxidin

Isofraxidin is a coumarin compound found in traditional Chinese herbs (Majnooni et al., 2020) and has been clearly shown by previous studies to have strong anti-inflammatory activity (Lin et al., 2018; Chen et al., 2020). Su et al. found that isofraxidin alleviated the IL-1β-induced upregulation of inflammatory mediators and cytokines (Su et al., 2019). In NP ECM metabolism, it can inhibit the expression of the ECM-degrading enzymes and promote the expression of type II collagen and aggrecan. In terms of the molecular mechanism, isofraxidin can inhibit the nuclear translocation and phosphorylation of p65, indicating that the NF-κB pathway is involved in the anti-inflammatory effect of isofraxidin. These studies show that isofraxidin can be used for treating IDD.

### Higenamine

Higenamine was extracted initially from the traditional Chinese herb aconite root in 1976 and later was identified as the main active component of many Chinese herbs (Ha et al., 2012). Higenamine has been found to have many biological activities (Bai et al., 2019; Zhang et al., 2019; Romeo et al., 2020; Yang et al., 2021), such as anti-inflammatory and antioxidant. Bai et al. found that the IL-1β-induced iNOS, PGE2, COX-2, TNF-α, and IL-6 levels, were attenuated by higenamine in NP cells (Bai et al., 2019). Moreover, Bai et al. have also found that higenamine suppressed the IL-1β-induced activation of the NF-κB signaling pathway.

### Sesamin

*Sesamum indicum* (sesame) is often used as a source of spices and edible oil. Sesamin is a type of sesame lignans that can be extracted from sesame oil (Dalibalta et al., 2020). Many studies have shown that sesamin has potential anti-inflammatory, antioxidant, and anti-tumor effects in different tissues (Majdalawieh et al., 2017; Dalibalta et al., 2020). The role of sesamin in IDD development has also been confirmed. The anti-inflammatory effects of sesamin on rat IVD have been examined by Li et al. The expression of inflammation factors and the migration of macrophages can be suppressed by sesamin treatment (Li et al., 2016). Subsequently, the inhibition of MAPK pathway activation was involved in its anti-inflammatory effect. In addition, Li et al. proved the inhibitory effects of sesamin on the occurrence and development of IDD through *in vivo* experiments (Li and Lv, 2020). These results suggest that sesamin can reverse the process of IDD by inhibiting inflammation.

### Honokiol

Honokiol (HKL) is a natural compound extracted from the roots and bark of *Magnolia* trees (Prasad and Katiyar, 2016). Previous studies have shown that it has apparent antagonistic effects on oxidative stress, inflammation, and tumor, and hence has been reported to be used in disease treatment (Chiu et al., 2021; Liu Y. et al., 2021; Lu et al., 2022). Tang et al. demonstrated that HKL inhibited the expression of NP cell apoptosis-related proteins induced by H_2_O_2_, the production of oxidative stress marker molecules, the level of inflammatory mediators, the expression of major extracellular matrix-degrading proteases, and then enhanced the expression of ECM anabolic proteins (Tang et al., 2018). The molecular mechanism of the action of HKL involves the inhibition of NF-κB/JNK signaling and TXNIP/NLRP3/caspase-1/IL-1β activation. SIRT3 is an important deacetylation modifying enzyme in mitochondria and is important for mitochondrial health. Wang et al. reported that HKL induced the upregulation of SIRT3 through the AMPK-PGC-1α axis, which improves the activity of antioxidant enzymes in mitochondria, and further prevents oxidative damage toNP cells (Wang et al., 2018b). *In vivo* experiments also confirmed the results of *in vitro* experiments. Therefore, HKL has the potential to treat IDD.

### Salvianolic Acid B

*Salvia miltiorrhiza Bunge*, also called Danshen, is atraditional Chinese herb. It has been used in Chinafor centuries (Wu et al., 2020; Xiao et al., 2020). Salvianolic acid B (SAB) is an abundant active ingredient from Danshen. SAB has been proved to have antioxidant (Zhao et al., 2019; Xiao et al., 2020) and anti-inflammatory activities (Ho and Hong, 2011). Dai et al. found that SAB slowed down the process of IDD and reconstructed the structure of IVD through *in vivo* experimental study. Subsequently, it was also confirmed that the levels of GSH and SOD2 in the degenerative IVD were reduced, and SAB treatment significantly reversed this change (Dai S. et al., 2021). In additional *in vitro* experiments, SAB was found to reduce the levels of ROS and MDA and increase the levels of GSH and SOD2. JAK2/STAT3 signaling pathway is suggested to be related to the antioxidant effect of SAB.

### Polydatin

Polydatin is the abundant form of resveratrol found in nature, and its average concentration in *Polygonum cuspidatum*andred wine is about 10times that of resveratrol (Liu W. et al., 2017). Polydatin, like resveratrol, has anti-inflammatory and antioxidant activities. It is worth noting that, unlike resveratrol, polydatin can enter cells through an active mechanism using glucose carriers and has a stronger anti-enzymatic oxidation ability than resveratrol. These properties allow polydatin to exhibit a higher absorption and better bioavailability relative to resveratrol (Tang, 2021). Wang et al. found that polydatin reduced the production of ROS through theNrf2 signaling pathway and protected rat NP cells from TNF-α-induced mitochondrial dysfunction and ECM degradation (Wang et al., 2018a). Moreover, Kang et al. found that polydatin upregulated Parkin and Nrf2 pathways, protecting CEP cells from H_2_O_2_-induced mitochondrial dysfunction, oxidative stress, and apoptosis, thereby inhibiting the development of IDD (Kang et al., 2020).

### Naringin

Naringin is a bioflavonoid found in the tangerine peel (Lavrador et al., 2018). It reportedly has a wide range of pharmacological activities, including antioxidant (Long et al., 2020; Bao et al., 2022) and anti-inflammatory effects (Zhao et al., 2020; Wu et al., 2021). Naringin has been confirmed to increase autophagy flux by activating the AMPK/SIRT1 pathway, thereby protecting NP cells from inflammatory response, oxidative stress, and impaired cellular homeostasis (Chen R. et al., 2022). Naringin can be developed into an effective drug to treat IDD.

### Baicalein

Baicalein is a flavonoid compound naturally found in the Chinese herb *Scutellaria baicalensis*. Baicalein possesses several pharmacological activities, including alleviation of inflammation (Wang X. et al., 2021; Jiang et al., 2022) and oxidative stress (Dai C. et al., 2021; Liu BY. et al., 2021). Baicalein helps treat IDD mainly *via* countering inflammation. It can inhibit NO, PGE2, TNF-α, and IL-6 induced by IL-1β (Jin et al., 2019). At the same time, it can reduce the expression of degrading enzymes MMP-13 and ADAMTS5 and upregulate the expression of aggrecan and type II collagen. A study examining its mechanism found that baicalein inhibited NF-κB and MAPK pathways. *In vivo* experiments also showed that baicalein could inhibit the process that led to IDD.

### Berberine

Berberine (BBR) is an isoquinoline alkaloid found in the long-used traditional Chinese herbs *Rhizomacoptidis, Cortex Phellodendri, and Mahonia bealei.* BBR reportedly has anti-inflammatory (Li Y. et al., 2022; Dai et al., 2022) and antioxidant properties (Cao et al., 2021; Seth et al., 2021). BBR has therapeutic effects on various diseases, including osteoarthritis. Its therapeutic potential in IDD has also been tested. Luo et al. demonstrated that BBR could inhibit oxidative stress-induced cell damage by regulating ER stress and autophagy (Luo et al., 2019). *In vivo* studies have also yielded similar evidence, suggesting that BBR treatment can delay the IVD destruction process induced by puncture in the rat model. Lu et al. found that BBR can inhibit the upregulation of NP extracellular matrix-degrading enzymes and the downregulation of the key components of the matrix, and excessive apoptosis induced by IL-1β (Lu et al., 2019).

### Genistein

Genistein is an isoflavone primarily identified in *Glycine* max *(soybean)* extract, among many other sources, such as peanuts, green peas, and legumes. Genistein reportedly prevents various diseases, such as anti-inflammatory, reducing osteoporosis, improving obesity, and anti-tumor (Fuloria et al., 2022; Goh et al., 2022; Ji et al., 2022). Wang et al. found that genistein can activate the Nrf2-mediated antioxidant defense system in NP cells (Wang et al., 2019) and subsequently inhibit TBHP-induced apoptosis and ECM degradation.

### Acacetin

Acacetin is a flavonoid compound from *Saussurea involucrata plant* and *Damiana*. Acacetin has been found to have anti-inflammatory (Ren et al., 2020; Singh et al., 2020), antioxidant (Song et al., 2022; Wu et al., 2022), anti-cancer (Wang S. et al., 2020; Yun et al., 2021), anti-osteoporosis (Jin et al., 2021; Lin et al., 2022), anti-diabetic (Han et al., 2020; Song et al., 2022) and other properties. Acacetin reportedly inhibits TBHP-induced ROS production by upregulating the expression of antioxidant proteins such as HO1, NQO1, and SOD in NP cells (Wang H. et al., 2020). Acacetin can also reduce the expression of inflammatory mediators such as COX-2 and iNOS induced by TBHP and also inhibits the degradation of extracellular matrix in NP cells. The activation of the Nrf2 pathway and the inhibition of the MAPK pathway are the specific mechanisms of the biological action of Acacetin. *In vivo* experiments also confirmed that Acacetin can reduce the process of IDD induced by puncture.

### Wogonin

Wogonin is an important flavonoid, isolated from the root of *Scutellaria baicalensis Georgi* (Chirumbolo, 2013)*.* It has many pharmacological effects, including antioxidant and anti-inflammatory (Zheng et al., 2020; Feng et al., 2022; Shao et al., 2022). Fang et al. found that wogonin can inhibit IL-1β-induced inflammatory response and extracellular matrix degradation in NP cells (Fang et al., 2018). Further studies revealed that wogonin playsa key role by activating Nrf2/HO-1 pathway and inhibiting MAPK signaling pathway.

### Luteoloside

Luteoloside, a type of flavonoid glycoside, can be isolated from the plant *Lonicera japonica* (Xiong et al., 2013; Lin et al., 2019; Shi et al., 2020). It has recently been reported to possess anti-inflammatory and antibacterial properties. Lin et al. found that luteoloside inhibited IL-1β-induced the level of TNF-α, iNOS, NO, IL-6, COX-2, and PGE2 in rat NP cells (Lin et al., 2019). The level of apoptosis and ECM degradation were also improved by luteoloside (Lin et al., 2019). In addition, luteoloside has been proved to play a role by activating the Nrf2 pathway and then inhibiting the NF-κB pathway.

### Quercetin

Quercetin is a type of natural flavonoid isolated from various fruits and vegetables (Mirazimi et al., 2022). Previous studies have shown that quercetin possesses many functions, including anti-cancer, anti-oxidative, and anti-inflammatory properties (Pinheiro et al., 2021; Yin et al., 2021; Sun et al., 2022). Wang et al. found that quercetin can activate the autophagy pathway through SIRT1 so as to reduce the level of ROS and alleviate apoptosis and extracellular matrix degeneration (Wang D. et al., 2020). These findings hint at a new and effective treatment for IDD.

## Summary and Prospect

Oxidative stress and inflammation play an important role in the progression of IDD. Elevated levels of ROS and increased production of inflammatory cytokines in the degenerative discs can activate multiple signaling pathways that cause damage to IVD cells, resulting in structural damage and dysfunction of IVD, which in turn leads to the development of IDD (Johnson et al., 2015; Feng et al., 2017; Cao et al., 2022). Therefore, using oxidative stress and inflammation as therapeutic targets for IDD has wide-ranging prospects. In recent years, the research and application of phytochemicals have attracted significant attention. This review revealed that various phytochemicals could exert inhibitory effects on IDD based on the data from *in vitro* and *in vivo* experiments, mainly by inhibiting oxidative stress and inflammation. Also, given the cost-effectiveness and the availability of phytochemicals and their therapeutic role in IDD, research on the use of phytochemicals to improve IDD is rapidly increasing. Phytochemicals have emerged as an important source for developing therapeutic agents for IDD. Future research needs to consider several key points. The first is that phytochemicals can often have multiple targets; therefore, the synergistic effects between multiple signaling pathways and the broad range of cellular functions involved must be analyzed in an integrated manner when designing therapeutic agents for IDD to minimize side effects and expand therapeutic effects. Secondly, most of the research on the role of phytochemicals in IDD treatment is to deliver drugs to animals through IVD injection and other methods. However, very few studies have focused on the metabolism and distribution of phytochemicals in animals. Due to the extensive distribution of phytochemicals in animals, side effects may be encountered in other organs apart from the therapeutic effect on IDD. Exploring the panorama of the metabolic processes and organ distribution of phytochemicals in animals can help select appropriate phytochemicals and delivery methodsso as to improve the therapeutic efficacy of phytochemicals on IDD. Additionally, the dose of phytochemicals also needs to be focused upon in future studies. Currently, many *in vitro* experiments are focusing on the therapeutic effect and toxicity of different doses of phytochemicals on IDD models. In contrast, little attention is paid to the therapeutic effect of different doses and long-term toxicity to animals *in vivo* experiments. Therefore, in future studies, it may be necessary to perform multiple-dose *in vivo* experiments to verify its therapeutic effect so as to evaluate the effect of phytochemicals more comprehensively. Finally, although many studies have uncovered the favorable effects of phytochemicals for treating IDD, a large number of clinical trial studies are needed to further confirm their effects when applying them to the treatment of patients. Researchers and experts need to work together to develop a systematic experimental design and experimental analysis and establish an effective evaluation system to evaluate phytochemicals more safely and rationally and make these phytochemicals more effective in benefiting patients with IDD.

## Abbreviations

LBP, low back pain; IVD, intervertebral disc; IDD, intervertebral disc degeneration; NP, nucleus pulposus; AF, annulus fibrosus; CEP, cartilage endplate; ECM, extracellular matrix; MMPs, matrix metalloproteinases; ADAMTS, a disintegrin and metalloprotease with thrombospondin motifs; VEGF, vascular endothelial growth factor; ROS, reactive oxygen species; AGEs, advanced glycation end products; CUR, curcumin; TBHP, Tert-butyl hydroperoxide; RES, resveratrol; EGCG, (-)-Epigallocatechin-3-gallate; CGA, chlorogenic acid; ICA, icariin; HKL, honokiol; SAB, salvianolic acid B; BBR, berberine.
